# Gut Microbiota and Type 1 Diabetes

**DOI:** 10.3390/ijms19040995

**Published:** 2018-03-27

**Authors:** Hui Han, Yuying Li, Jun Fang, Gang Liu, Jie Yin, Tiejun Li, Yulong Yin

**Affiliations:** 1 Key Laboratory of Agro-Ecological Processes in Subtropical Region, Institute of Subtropical Agriculture, Chinese Academy of Sciences, National Engineering Laboratory for Pollution Control and Waste Utilization in Livestock and Poultry Production, Changsha 410128, China; hanhui16@mails.ucas.ac.cn (H.H.); liyuying@mails.ucas.ac.cn (Y.L.); gangle.liu@gmail.com (G.L.); yinyulonsg@isa.ac.cn (Y.Y.); 2 University of Chinese Academy of Sciences, Beijing 100039, China; 3 College of Bioscience and Biotechnology, Hunan Agricultural University, Changsha 410128, China; fangjun1973@hunau.edu.cn; 4 Hunan Co-Innovation Center of Animal Production Safety, Changsha 410128, China

**Keywords:** gut microbiota, type 1 diabetes, diet, immune response, hormones

## Abstract

Recently, the onset of type 1 diabetes (T1D) has increased rapidly and became a major public health concern worldwide. Various factors are associated with the development of T1D, such as diet, genome, and intestinal microbiota. The gastrointestinal (GI) tract harbors a complex and dynamic population of microorganisms, the gut microbiota, which exert a marked influence on the host homeostasis and metabolic diseases. Recent evidence shows that altered gut bacterial composition (dysbiosis) is highly associated with the pathogenesis of insulin dysfunction and T1D and, thus, targeting gut microbiota may serve as a therapeutic potential for T1D patients. In this study, we updated the effect of gut microbiota on T1D and potential mechanisms were discussed.

## 1. Gut Microbiota and Type 1 Diabetes (T1D)

Diabetes is becoming a common metabolic disease worldwide. Type 1 diabetes (T1D) is an autoimmune disease characterized by insufficient insulin production as a result of T-cell-mediated destruction of insulin-secreting pancreatic beta cells. The International Diabetes Federation estimated that more than 542,000 children suffered from T1D in 2015 and the occurrence is increasing by an annual rate of 3% with approximately 86,000 children developing T1D every year [1]. In T1D patients, deficiency of insulin impedes glucose uptake into tissues and the chronic accumulation of circulating glucose is highly associated with pathological injury, leading to the development of disabling and life-threatening health complications [2,3,4]. 

Various factors are involved in the development of T1D, including diet, genome, and gut microbiota [5,6,7]. Humans and animals have various microbial communities and the great majority of microbiota inhabit the gastrointestinal track. There are about 500–1000 species of gut microorganisms in humans [8], and the adult intestine consists of approximately 100 trillion bacterial cells, which is 10 times more than the number of human cells in the body itself [9]. Consumption of various nutrients rapidly and reproducibly can shape the structure and activity of gut microbiota, which in turn produce numerus molecules that are absorbed by the host and involved in various physiological processes. Carmody et al. tested the hypothesis that dietary intake dominated the interindividual variation in gut microbial community structure of mice [10]. On the other hand, gut microbiota is essential to its host development [8,11,12]. For instance, the gut microbial metabolites change dynamically and affect host health and disease states [13]. Alterations in gut microbial composition play an essential role in disease, such as heart failure, kidney disease, obesity, and diabetes mellitus [14,15,16,17]. Moreover, a number of studies indicate that gut microbiota is associated with T1D development [18,19,20]. Thus, in this study, we review the association between gut microbiota and T1D and discuss the potential mechanisms whereby gut microbiota exert influence on T1D development.

## 2. Gut Microbial Composition and T1D

Several studies have shown that the gut microbial composition differs between heathy hosts and hosts with T1D or at risk of T1D (Table 1). Bio-Breeding (BB) rat and non-obese diabetic (NOD) mouse exhibit similar characteristics to human disease [21]. In Bio-Breeding diabetes-prone (BB-DP) rats, before the onset of T1D, the composition of gut microbiota is markedly different between the rats that eventually will and will not develop T1D [18]. Similarly, Luiz et al. observed a significant decrease in the number of *Lactobacillus*, *Bryantella*, *Bifidobacterium*, and *Turicibacter* in BB-DP rats, whereas the number of *Bacteroides*, *Eubacterium*, and *Ruminococcus* increased in BB-DP rats compared with the Bio-Breeding diabetes-resistant (BB-DR) rats [22]. 

Consistent with animal models, the gut microbial composition is also different between humans with TID and healthy humans. In a case-control study that included 16 children with T1D and 16 healthy children, gut microbial composition showed marked differences between the healthy children and the children with T1D [23]. At the phylum level, the abundance of Actinobacteria and Firmicutes, and the ratio of Firmicutes to Bacteroidetes were all lower in the children with T1D than the healthy children [23]. At the genus level, the healthy children had greater numbers of *Lactobacillus*, *Bifidobacterium*, *Blautia coccoides*/*Eubacterium rectale* group, and *Prevotella* in the gut, whereas children with T1D contained greater numbers of *Clostridium*, *Bacteroides*, and *Veillonella* [23]. In addition to microbial abundance, the diversity and stability of intestinal microbiota are also associated with the development of T1D. In a four-matched case-control study, the microbiomes of healthy children were more diverse and stable when compared to children who developed autoimmunity and eventually T1D [24]. Meanwhile, the Firmicutes levels were decreased and the Bacteroidetes levels were increased as children developed autoimmune disorders [24]. Moreover, compared with individuals with preclinical T1D and healthy individuals, insufficient butyrate-producing bacteria and low bacterial diversity and community stability seemed to emerge after the appearance of T1D auto-antibodies [25]. Thus, the instability of microbiomes and high ratio of Firmicutes to Bacteroidetes may be early diagnostic markers of pending autoimmune disorders such as T1D [24]. A recent study has shown that daily intake of *Lactobacillus reuteri* increases the secretion of insulin and incretin in glucose-tolerant humans [26]. However, the effect may be caused by an improvement of insulin beta-cell function rather than an alteration of fecal microbiota [26]. Similarly, results from a study including 298 stool samples taken up to age 3 years indicated that there was no difference in gut microbial composition and diversity between anti-islet cell autoantibody-positive children and anti-islet autoantibody-negative children [27]. The authors then performed microbial interaction networks at the genus level by using two scores: eigenvector centrality (EC) and the number of isolated nodes. In a network, EC measures the centrality of its neighbors and node degree measures the connectivity in a topological sense. The results showed that anti-islet cell autoantibody-positive children had more nodes with intermediate levels of EC and isolated nodes than anti-islet cell autoantibody-negative children, which may decrease the number of possible communication paths, and thus cause a negative effect on the adaptability of the microbial community [27]. 

Taken together, the abundance, stability, and connectivity of gut microbiota are all associated with T1D development. This may be because low diversity and amounts of the intestinal microbiota limit their ability to digest a diverse diet, leading to a decreased number or diversity of gut microbial fermentation products, which may eventually cause metabolic diseases including diabetes. 

## 3. Gut Microbiota Influence T1D Development by Affecting the Immune Response 

T1D is believed to be a beta cell-mediated pro-inflammatory state induced by innate and adaptive immunity [28,29]. Toll-like receptors (TLRs) play an essential role in protecting the host from infectious microbes. TLRs recognize pathogen-associated molecular patterns (PAMPs) derived from microbiota [30]. Moreover, TLRs can induce the maturation of dendritic cells (DCs). Therefore, TLRs are responsible for innate and adaptive immunity [31]. Myeloid differentiation primary response gene 88 (MyD88) contributes to the downstream signaling pathways of TLRs [30,31].

An increasing number of studies have shown that gut microbiota is involved in the development of T1D via influencing immune response. Endotoxin, also known as lipopolysaccharide (LPS), is a major component of the outer membranes of Gram-negative bacterial species and plays an important role in increasing the level of proinflammatory cytokines and impairing pancreatic beta-cell function [32], which may lead to diabetes [33]. A case-control study showed that individuals with T1D had higher circulating LPS than nondiabetic individuals [34]. Moreover, LPS is believed to be derived from microbiota in the gut. Therefore, LPS may act as a molecular link between gut microbiota, inflammation, and T1D. In addition, gut microbial alteration can induce the leakage of LPS and fatty acids through destroying the intestinal mucosal barrier, which further activates TLR4 and results in metabolic inflammation [35]. In order to test the contributions of TLR4 to T1D development, Elke et al. generated a strain of NOD mouse that lacks the expression of TLR4 and assessed the development of diabetes in vivo and in vitro [29]. The results showed that deficiency of TLR4 accelerated the development of diabetes and enhanced the immune cell infiltration of islets in NOD mice [29]. Meanwhile, MyD88 knockout NOD mice were protected from the development of T1D when raised under normal specific-pathogen free (SPF) condition, whereas NOD mice lacking MyD88 protein robustly developed T1D when housed under germ-free (GF) condition [19]. They also observed that deficiency of MyD88 altered the gut microbial composition. To further assess the role of microbiota in T1D development, they established altered Schaedler Flora (ASF)-colonized NOD.MyD88KO mice. They found that ASF-colonized NOD.MyD88KO mice had decreased incidence of diabetes and islet infiltration when compared to GF NOD.MyD88KO mice. Hence, they suggested that the interaction of gut microbiota with innate immune system was responsible for the onset and progression of T1D [19]. Taken together, we hypothesize that gut microbiota affect the progression of T1D via the involvement of TLR4 and MyD88. Some studies have shown that gut microbiota is involved in influencing the development of T1D by affecting anti/pro-inflammatory interleukin levels. BB-DP rats fed with *L. johnsonii* had significantly lower level of interferon-gamma (IFN-γ) than diabetic rats [20]. Oral administration of probiotic bacteria to NOD mice showed increased interleukin-10 (IL-10) levels in the pancreas and lower incidence of T1D [36]. In BB-DP rats, antibiotic treatment decreased the abundance of gut microbiota, and reduced and delayed the onset of diabetes [18]. Furthermore, antibiotic treatment could modulate the function of pancreatic beta-cells. Thus, Brugman suggested that antibiotic treatment could influence the function of beta cells in the pancreas and eventually affect T1D development [18]. Recently, Mullaney et al. investigated the relationship between T1D genetic risk and intestinal microbiota [37]. Their findings indicated that T1D susceptibility alleles contribute toward shaping intestinal microbiota [37]. It is known that regulatory T (Treg) cells play an important role in protecting against the incidence of T1D [38]. They also found that low-dose treatment with IL-2 increased the number of Treg cells, reduced inflammatory response and protect NOD mice from developing diabetes. Moreover, IL-2 treatment decreased the number of Bcteroidales and Oscillospira, whereas it increased the number of *Bifidobacterium*. Meanwhile, they found that T1D-protective alleles within the IL-2 pathway are linked to reduced numbers of the Clostridiales, Bacteroides, Lachnospiraceae, Ruminococcaceae, and Rikenellaceae families in humans [38]. Thus, these data show that T1D susceptibility alleles contribute to the alterations of intestinal microbiota and immune response.

In addition, the intestinal epithelium acts as an important selective filter between the organism and the external environment. A healthy intestinal epithelium allows the translocation of water, nutrients, and bioactive compounds from the intestinal lumen into circulation [39]. An increasing number of studies indicate that in BB-DP rats, increased intestinal permeability allows unregulated passage of environmental antigens, which can induce the autoimmune response and subsequently promote the development of T1D [20,40]. Furthermore, previous studies have reported that oxidative stress response can disrupt the intestinal mucosal barrier and contribute to T1D development [41]. Valladares et al. found that administration of *L. johnsonii* N6.2 can maintain the intestinal barrier dysfunction by affecting the levels of tight junction genes [20]. Furthermore, *L. johnsonii* N6.2 feeding altered the gut microbial composition and delayed the onset of T1D in BB-DP rats [20]. On the other hand, some *Bacteroides*, *Bifidobacterium*, and *Ruminococcus* members have the ability to degrade mucins and subsequently reduce the integrity of the mucosal barrier [42]. Moreover, compared to diabetic rats, administration of *L. johnsonii* N6.2 to BB-DP rats decreased the levels of markers of oxidative stress, such as superoxide dismutase 2 (Sod2), catalase (Cat), glutathione reductase (GR), glutathione peroxidase (Gpx1), and inducible nitric oxide synthase [20]. Meanwhile, administration of *L. johnsonii* N6.2 upregulated the expression level of Cox-2, which may protect hosts against T1D. 

Mucosal-associated invariant T (MAIT) cells are innate-like lymphocytes that have a protective effect on bacterial infections. The abundance of MAIT cells in the thymus and spleen was lower in germ-free mice compared with specific-pathogen-free mice [43]. Similarly, compared with the adults not at risk of developing TID, the adults at risk of developing T1D had higher numbers of CD25^+^ and PD-1^+^ MAIT cells and lower numbers of CCR6^+^ MAIT cells [43]. Additionally, the MAIT cells are localized preferentially in mucosal tissues, like the intestine. Thus, there may be an interaction between MATI cells and intestinal microbiota. MAIT cells are known to participate in beta-cell death. Thus, MAIT cells may play a role in the incidence of T1D. Koay et al. analyzed the cytokine production of MAIT cells to explore the effect on T1D and the results showed that the amounts of IFN-R and GzB produced by MAIT cells are higher in mice with diabetes than pre-diabetic mice, which may lead to death of pancreatic beta-cells [43]. Thus, these data demonstrated that MAIT cells have a role in the development of T1D by promoting pancreatic beta-cell death. They also suggested that MAIT cells increase the permeability of the gut, which may alter the translocation of bacteria components (including 16S bacterial DNA) from the intestine to peripheral tissues [43]. As a result, the bacterial components contribute to the development of T1D via stimulating the pancreatic lymph node (PLN) dendritic cells and promoting anti-islet T cell responses [43]. 

Collectively, these experiments support the notion that gut microbiota impact T1D development by altering intestinal permeability and immune response.

## 4. Hormones, Gut Microbiota, and T1D 

Hormones and gut microbiota are associated with autoimmune disease and they may affect immune response through shared pathways or additive effects [44]. Some studies show that female NOD mice develop more T1D than males, which may link with hormonal difference and microbiota. Yurkovetskiy et al. observed that female NOD mice have higher incidence of T1D than male NOD mice when fed under SPF conditions [45], which is consistent with the research conducted by Markle et al. [46]. However, such gender bias was abolished when the mice were fed under GF conditions [45]. They also found that post-pubescent male and female mice had different gut microbial communities [46]. In addition, IFN-γ and IL-1ß were involved in the gender bias in the TID development of NOD mice. In their study, blood androgen concentration was not involved in protecting male NOD mice from T1D, but they suggested that hormone and selected microbial lineages provide a regulatory feedback loop, which was associated with the gender bias. Markle et al. found that microbial colonization influenced sex hormone levels [45]. Moreover, transplantation of gut microbiota from adult male NOD mice to immature female NOD mice changed the recipient’s gut microbiota and testosterone levels and resulted in T1D protection. Collectively, these data demonstrate that sex hormones alter the gut microbiota and the immune response and eventually influence the incidence of T1D.

## 5. Diet, Gut Microbiota, and T1D

Previous studies have indicated that diet rapidly alters the gut microbial composition and affects T1D development in humans and animals [47]. In our study, we found that dietary glutamine can affect gut microbiota and eventually influence the intestinal immunity [48,49]. However, a study that included 2520 youth with T1D showed that four dietary quality approaches (including dietary approaches to stop hypertension, healthy eating-index 2010, modified KIDMED, and total antioxidant capacity) failed to affect the biomarkers of inflammation (C-reactive protein, fibrinogen, and IL-6) [50]. More recently, we found that lysine restriction affects the gut microbiota and inflammatory response of piglets [51,52]. The data demonstrated that when fed a diet containing 70% lysine of the control, the ileal microbial diversity tended to increase in piglets compared with the 100% lysine group, although the changes were insignificant [51]. Moreover, in the control group, once the 100%-lysine diet changed to a 70%-lysine diet, the microbial diversity was significantly increased [51]. However, in the 70%-lysine group, when the diet changed to a 100%-lysine diet, the microbial diversity was significantly decreased [51]. Lysine restriction also influenced the gut microbial composition at the phylum and family levels [51]. Gluten is a complex molecule and has a high abundance of glutamine and proline, which make them difficult to be digested in the intestine. Gluten intake can lead to coeliac disease (CD) and may also influence the development of T1D [53,54]. Patients diagnosed with CD at a young age have shown an increased risk of developing an autoimmune disease. Meanwhile, in adults, a gluten-free diet plays a role in preventing the development of autoimmune disease [55]. A study in NOD mice showed that gluten-free diet decreased numbers of caecal bacteria and Gram-positive bacterial when compared to a standard diet containing wheat proteins [56]. Meanwhile, mice with diabetes have a higher number of caecal bacteria than mice without diabetes. Furthermore, Gram-positive bacteria influence the digestive production and immune response, which therefore contribute to the development of diabetes. Similarly, Marietta et al. conducted a study to investigate the effect of glute intake on diabetes incidence. The results showed that compared with a gluten-containing diet, a gluten-free diet can delay the onset and decrease the incidence of T1D in NOD mice and increase the numbers of T regulatory cells. NOD mice fed with a gluten-free diet have decreased numbers of *Bifidobacterium*, *Tannerella*, and *Barnesiella* species, and an increased number of *Akkermansia* in the intestine, compared with NOD mice fed with a gluten-containing diet. Interestingly, after adding gluten to the gluten-free diet, the numbers of *Bifidobacterium*, *Tannerella*, and *Barnesiella* species increased and the number of *Akkermansia* decreased. Thus, gluten intake seems to directly modulate the intestinal microbial composition and affect the development of T1D [57]. These data demonstrate that gluten-free diet plays a role in mediating the function of beta-cells by changing the intestinal microbiota, which may influence the incidence of diabetes. Furthermore, some studies have shown that the effect of gluten on the development of T1D is associated with the amount, timing, and mode of gluten intake [58,59,60]. Previous studies have shown that dietary gluten reduced the onset of T1D via altering the gut microbial composition and the immune response [53,57]. One study suggested that treatment with dietary fermentable fibers could promote the development of T1D by elevating the abundance of Bacteroidetes phylum in the intestine [61]. In another study, the results showed that high-fat-diet-treated mice had significantly lower numbers of *Bifidobacterium* species and higher intestinal endotoxin than the control mice [62]. Meanwhile, high-fat-diet-treated mice showed increased plasma pro-inflammatory cytokines, strong glucose intolerance, and decreased insulin secretion compared with the control mice. Therefore, the authors tested whether *Bifidobacterium* was involved in modulating intestinal endotoxin, inflammatory response, and the development of diabetes. They specifically increased the abundance of gut *Bifidobacterium* by feeding the high-fat-diet-treated mice with a prebiotic. The results showed that the administration of a prebiotic could increase the abundance of *Bifidobacterium*, decrease intestinal endotoxin, improve glucose tolerance, restore the insulin secretion, and alleviate the inflammatory response compared with the high-fat-diet-treated mice [62]. It is therefore evident that a high-fat diet may cause metabolic diseases (including diabetes) via altering the composition of gut microbiota, particularly decreasing *Bifidobacterium*. Furthermore, the administration of specific prebiotics could protect against the metabolic disorders induced by high-fat diet. Thus, altering the composition of gut microbiota specifically may be an effective means in which to alleviate the development of diabetes. 

In addition, a recent study showed that diet has an impact on gut microbial metabolites [63]. Gut microbiota ferment the dietary polysaccharides and produce monosaccharides and short-chain fatty acids (SCFAs). The SCFAs are absorbed by the colon epithelium, which may have an effect on metabolic response, immune response, and inflammatory disease [64,65]. For instance, acetate and propionate can influence insulin sensitivity and glucose tolerance through mediating glycemic response. Butyrate, also one of the SCFAs, maintains the integrity of the gut epithelium by inducing mucin synthesis [66] and improves the intestinal barrier by facilitating tight junction assembly [67], which is involved in autoimmune diseases [68], such as T1D. However, other SCFAs do not improve gut integrity [69]. Sun et al. found that NOD mice had lower abundance of SCFAs, especially butyrate, when compared to the healthy group [70]. Meanwhile, injection of butyrate decreased the onset of diabetes of prediabetic NOD mice. Sodium butyrate decreased cathelicidin gene expression in human lung epithelial cell [71], which is associated with T1D [72]. Furthermore, compared to the children who eventually developed T1D, the butyrate-producing bacteria were more abundant in the control group [68]. It is therefore conceivable that the ratio of butyrate-producing to other SCFA-producing gut bacteria play a role in T1D. 

It should be noted that there are more than three billion people suffering from various types of micronutrient deficiencies, particularly vitamin A, iron, and zinc. Though conventional diets play an important role in shaping and altering gut microbiota, the individual effects of minor food components should also receive attention. Vitamin A deficiency increases the ratio of Firmicutes/Bacteroidetes and decreases the level of butyrate-producing bacterial, which may protect hosts against the development of various chronic diseases such as diabetes [73]. In addition, vitamin A metabolite retinoic acid has the ability to inhibit the differentiation of pro-inflammatory Th17 cells induced by IL-6 and promote the differentiation of anti-inflammatory Treg cells, which may contribute to preventing the incidence of T1D [74,75]. Iron fortification decreases *Lactobacilli* and increases Enterobacteria in African children [73]. Supplementation of zinc can increase bacterial diversity, richness, and activity in the ileum of piglets [76,77,78]. Moreover, zinc deficiency may promote the development of T1D via affecting the inflammatory response and impacting the metabolic control [79]. In addition, prebiotics via food additives elevate protective intestinal microbiota and improve the permeability of the intestinal barrier, thus helping to improve health and prevent the development of diet-related chronic diseases including diabetes [80,81]. Some selected prebiotics inhibit the glycemic response to maltose or sucrose by decreasing the activity of disaccharidase in the gut [81]. Additionally, the administration of prebiotics significantly alters the gut microbial composition, improves intestinal permeability, and decreases inflammatory response, which may alleviate the development of diabetes [81]. In addition, prebiotics are fermented by gut microbiota and produce propionate. Propionate can decrease fasting blood glucose, reduce gluconeogenesis, and promote the utilization of glucose [81]. Meanwhile, it has been shown that propionate may elevate the levels of glucagon-like peptide and peptide YY. It is therefore evident that prebiotics improve glycemic control via their fermentation by gut microbiota. Non-nutritive sweetener is a widely used food additive. It can promote the development of glucose intolerance via influencing the composition and function of intestinal microbiota [82], which may impair glucose homeostasis and eventually lead to T1D [83].

Together, these findings indicate that conventional diets, minor food components, and food additives can alter the end-products of microbial fermentation and immune response by influencing intestinal microbial composition and activity, and may eventually play an important role in the development of T1D. Therefore, diabetes is a multisystemic disorder and as a preventive/protective measure, individuals should ensure that a healthy balance of dietary nutrients is obtained [84]. 

## 6. Conclusions

A mounting number of studies have shown that gut microbiota is associated with host health and disease. First, factors such as the conventional diet, minor food components, and hormones are involved in the development of T1D as outcomes of their influence on gut microbial composition, stability, and connectivity. Second, gut microbiota can alter the development of T1D by influencing the immune response of hosts. These studies help us to understand the underlying mechanism of T1D development and provide evidence that will support the discovery of effective treatments for T1D. For example, the application of new harmless wheat, acetate, and/or butyrate may reduce the onset of T1D [12,85]. However, there are still some questions that require further research, such as the specific molecular mechanisms by which gut microbiota affects the T1D development and potential treatments to T1D through altering gut microbiota and diet.

## Figures and Tables

**Table 1:** Changes in gut microbiota of different models with type 1 diabetes or at risk of type 1 diabetes.

Models	Changes in Gut Microbiota	Reference
Bio-Breeding diabetes-prone rats and Bio-Breeding resistant rats	Increased *Bacteroides*	[18]
Bio-Breeding diabetes-prone rats and Bio-Breeding resistant rats	Decreased *Lactobacillus*, *Bryantella*, *Bifidobacterium*, and *Turicibacter* and increased *Bacteroides*, *Eubacterium*, and *Ruminococcus*	[22]
16 children with type 1 diabetes and 16 healthy children	Decreased Actinobacteria and Firmicutes levels, decreased ratio of Firmicutes to Bacteroidetes, and increased Bacteroidetes; Decreased *Lactobacillus*, *Bifidobacterium*, *Blautia coccoides*/*Eubacterium rectale* group and *Prevotella* and increased *Clostridium*, *Bacteroides*, and *Veillonella*	[23]
4 matched case-control in children	Decreased Firmicutes and increased Bacteroidetes	[24]
